# Screen for Footprints of Selection during Domestication/Captive Breeding of Atlantic Salmon

**DOI:** 10.1155/2012/628204

**Published:** 2012-12-27

**Authors:** Anti Vasemägi, Jan Nilsson, Philip McGinnity, Tom Cross, Patrick O'Reilly, Brian Glebe, Bo Peng, Paul Ragnar Berg, Craig Robert Primmer

**Affiliations:** ^1^Division of Genetics and Physiology, Department of Biology, University of Turku, 20014 Turku, Finland; ^2^Department of Aquaculture, Institute of Veterinary Medicine and Animal Science, Estonian University of Life Sciences, 51014 Tartu, Estonia; ^3^Department of Wildlife, Fish, and Environmental Studies, Swedish University of Agricultural Sciences, 901 83 Umeå, Sweden; ^4^Aquaculture and Fisheries Development Centre, School of Biological, Earth, and Environmental Sciences, University College Cork, Cork, Ireland; ^5^Marine Institute, Furnace, Newport, Co. Mayo, Ireland; ^6^Population Ecology Division, Department of Fisheries and Oceans, Bedford Institute of Oceanography, Challenger Drive, Dartmouth, NS, Canada B2Y 4A2; ^7^Fisheries and Oceans Canada, Department of Fisheries and Oceans, St. Andrews Biological Station, St. Andrews, NB, Canada E0G 2X0; ^8^Department of Genetics, The University of Texas M. D. Anderson Cancer Center, Houston, TX 77030, USA; ^9^Department of Animal and Aquacultural Sciences, Centre for Integrative Genetics, Norwegian University of Life Sciences, 1432 Aas, Norway

## Abstract

Domesticated animals provide a unique opportunity to identify genomic targets of artificial selection to the captive environment. Here, we screened three independent domesticated/captive Atlantic salmon (*Salmo salar*) strains and their wild progenitor populations in an effort to detect potential signals of domestication selection by typing of 261 SNPs and 70 microsatellite loci. By combining information from four different neutrality tests, in total ten genomic regions showed signs of directional selection based on multiple sources of evidence. Most of the identified candidate regions were rather small ranging from zero to a few centimorgans (cM) in the female Atlantic salmon linkage map. We also evaluated how adaptation from standing variation affects adjacent SNP and microsatellite variation along the chromosomes and, by using forward simulations with strong selection, we were able to generate genetic differentiation patterns comparable to the observed data. This study highlights the significance of standing genetic variation during the early stages of adaptation and represents a useful step towards identifying functional variants involved in domestication of Atlantic salmon.

## 1. Introduction

Over the last decade, advances in genomic technologies together with developments in methods of statistical analyses have resulted in considerable progress in the systematic detection of genomic regions influenced by artificial selection [1–5]. These studies rely on the concept of genetic hitchhiking which predicts that selection affects the genome in a locus-specific manner by leaving specific signatures surrounding the gene or genes under selection [6]. Specifically, the theory of genetic hitchhiking focuses on the spread of novel advantageous mutations in a population, generating characteristic footprints of selection [7, 8]. This type of selection is often referred to as a “hard sweep” in contrast to the “soft sweep”, where selection acts on a standing variation [9]. The importance of hard sweeps versus soft sweeps in natural populations and domesticated strains remains an open question, although recent empirical work [10, 11] and theoretical models suggest that soft sweeps are likely to be widespread [9, 12, 13]. Nevertheless, hitchhiking-mapping efforts in domesticated animals and plants to date have mainly focused on identifying relatively old footprints of selection dating back hundreds or thousands of generations, during which time new advantageous mutations can spread to fixation (e.g., [1, 3, 14]) and very few studies have specifically focused on genetic mechanisms of the early stages of domestication (e.g., [15]).

Atlantic salmon (*Salmo salar* L.) represents a useful model for studying the genetic basis of recent, ongoing domestication since reared salmon strains have experienced intense artificial selection for only a short period of time (5–12 generations), and there are still many wild populations available for comparative purposes, including progenitor populations. The traits that have been targets of selective breeding in reared Atlantic salmon strains include higher growth rate, delayed sexual maturity, bacterial resistance, flesh colour, and fat content. The selection response of many of these traits has been as high as 10% per generation [16]. As a result, despite the short history of domestication, reared strains of Atlantic salmon often differ markedly from their wild counterparts [17]. In addition to farming, Atlantic salmon has also been a popular target for various restoration and supplementation/stocking programmes (here termed ranching), in which the species is captively bred and reared for subsequent release into the natural environment. Importantly, artificial propagation and rearing processes in the hatchery environment can involve intentional and/or inadvertent selection and relaxation of natural selection operating in the wild [18]. Inadvertent (nondeliberate) selection during captive breeding is particularly problematic for supportive breeding of endangered populations because it is essentially unavoidable and often associated with a reduction in fitness in the natural environment [19–21]. Importantly, the negative effects of domestication can be severe and even a few generations of captive breeding may have marked effects on natural reproduction in the wild [21–23]. 

Earlier studies on Atlantic salmon have demonstrated that 5–7 generations of domestication were sufficient to induce heritable changes in gene transcription level between domesticated and wild populations [24, 25]. Large proportion of these changes has been population specific while small number of genes has also shown to exhibit parallel changes in gene expression level in multiple wild-farmed population comparisons [24, 25]. The presence of parallel changes in transcription level may indicate (i) that the same DNA sequence polymorphisms are responsible for the rapid changes in gene expression, or alternatively, (ii) that the same downstream pathways, involving the same or different DNA sequence polymorphisms, are being affected during the domestication process. However, currently very little is known about what genomic regions have been influenced by domestication and captive breeding in different Atlantic salmon populations. Here, we screened 261 SNPs and 70 microsatellite loci in three independent-reared strains (two of which were used for ranching (IRL, SWE) and one for farming (CAN)) and their wild progenitor populations of Atlantic salmon to identify recent footprints of selection related to domestication and captive breeding. 

## 2. Materials and Methods

### 2.1. Study Populations

Three independently reared strains and their wild progenitor populations from Europe and North America were used in the analyses: Burrishoole River (Mayo, Ireland, IRL), Ume/Vindelälven Rivers (Västerbotten, Sweden, SWE), and the Saint John River (New Brunswick, Canada, CAN), in an attempt to identify genomic regions affected by domestication/captive breeding. A short description of the history of each reared strain and sampling details are provided below.

Artificial rearing of juvenile salmon for ranching from the Burrishoole river began in 1960 and recruitment from the wild population continued until 1964. Apart from the addition of a small number of wild fish in the reared broodstock between 1970 and 1975, the breeding strain has been effectively closed since that time; the hatchery born fish were distinguished from their wild counterparts by the absence of a previously-clipped adipose fin. Assuming a generation time of 5 years for the wild fish, wild and reared (ranched) Burrishoole salmon have experienced different selection pressures for approximately 8 generations during the early freshwater phase (from fertilization to smoltification). Today, the Burrishoole-ranched strain is composed of three almost distinct coexisting cohorts with little overlap between year classes [23]. Burrishoole wild samples consisted of 16 individuals collected as descending smolts in 2006, while the samples from the reared (ranched) Burrishoole strain were obtained from the smolts collected in 2005 and 2006 (eight individuals per cohort).

The wild River Umeälven salmon were extirpated by power plant dam construction in the early 1950s but River Vindelälven (a tributary of Umeälven) continued to support wild salmon. The present hatchery stock of River Umeälven originates from the wild Vindelälven population and since 1971, all hatchery (ranched) smolts have had their adipose fin removed. Since 1974 all salmon without adipose fins (hatchery fish) or with intact adipose fins (wild salmon) have been recorded at the fish trap below the Norrfors hydroelectric dam and only salmon of wild origin are released above the dam to continue their upstream spawning migration to the Vindelälven. As a result, wild and captive (ranched) Ume/Vindelälven salmon have experienced different selection pressures for approximately 5-6 generations during the early freshwater phase (from fertilization until smoltification) assuming a generation time of 5-6 years. River Ume/Vindelälven wild samples consisted of 16 returning individuals in 2006, while the samples from the reared (ranched) strain were obtained in 2009 (16 1+ old juveniles).

The Saint John reared strain used for farming was founded in 1990 from wild Saint John River salmon, and in contrast to IRL and SWE populations, by 2006 it has experienced four generations of selection for higher growth and reduced early maturation [30]. As a result, wild and reared salmon from Saint John river have experienced different selection pressures due to selective breeding relative to wild salmon from the same system, for approximately 4-5 generations. The Saint John River sample collection analyzed here consisted of 16 returning adults of wild origin collected in 2000 (Nashwaak tributary, situated below Mactaquac Dam), while the samples from the Saint John river aquaculture strain came from Atlantic Salmon Broodstock Development Program (ASBDP) of New Brunswick (randomly selected breeders from the selection line SGRP 90 JC, sampled in 2007).

### 2.2. SNPs Genotyping

In total, 320 SNP developed by Moen et al. [28] were genotyped using Sequenom MassArray TM system from (San Diego, USA). After quality control and assessment of levels of polymorphism, 59 markers were excluded from further analyses leaving a final set of 261 SNPs upon which all analyses were based (see Supplementary Material avaliable online at doi:10.1155/2012/628204). Most of these SNPs were initially mapped by Moen et al. [28] and later many of them have been incorporated into a larger Atlantic salmon SNP linkage map [31]. The linkage group information used throughout the study corresponds to the SALMAP consortium nomenclature [32] while information about the corresponding chromosome numbers was derived from Phillips et al. [33]. Genotyping was performed using Sequenom iPLEX Gold chemistry according to the manufacturer's instructions. Detailed information about PCR conditions, PCR and extension primers, and post-PCR pooling procedures are similar to Moen et al. [28] and available on request. Genotypes were assigned in real time [34] using the MassARRAY SpectroTYPER RT v3.4 software (Sequenom) based on the mass peaks present. All results were manually inspected using the MassARRAY TyperAnalyzer v3.3 software (Sequenom).

### 2.3. Microsatellite Genotyping

A total of 70 variable number tandem repeat markers were also included in this study. They included two MHC linked mini- and microsatellite loci, 21 EST-derived microsatellite loci [35, 36], and 47 anonymous microsatellite loci (Supplementary Material). The microsatellite markers were PCR amplified in 19 multiplex reactions that were subsequently combined into six capillary electrophoresis runs. All reactions were carried out in 6 *μ*L volumes including 10–100 ng of genomic DNA and 0.033–0.3 *μ*M of locus specific primers, the forward primer being labelled with one of four fluorescent dyes (four colour set: PET, FAM, NED, and VIC, three colour set: FAM, HEX and NED), and 1 × QIAGEN multiplex PCR master mix. The thermal cycling conditions used were as follows: 15 min initial activation step at 95°C followed by 35 cycles of denaturation at 94°C for 30 s, annealing at 55–60°C for 90 s and extension at 72°C for 60 s and ending with a final extension at 60°C for 10–30 min. Amplifications were performed using an Applied Biosystems 2720, a PTC-100, or a PTC-200 (Bio-Rad) thermal cycler. The PCR products from separate multiplex reactions were pooled (1–2.5 *μ*L) and made up to the final volume of 120 *μ*L of distilled water, mixed with GS600LIZ size standard (Applied Biosystems), and formamide for electrophoresis on an ABI 3130 × l automated sequencer. Detailed multiplex amplification information, including specific primer concentrations, annealing temperatures, and post-PCR pooling procedures, are available on request.

### 2.4. Summary Statistics

Diversity indices were estimated using the program FSTAT 2.9.3 [37]. Deviations from Hardy-Weinberg equilibrium were tested with the exact test [38] as implemented in GENEPOP 3.4 [26]. Genetic differentiation between populations was measured with pairwise *F*_ST_ estimates [27]. The significance of *F*_ST_ estimates between pairwise comparisons was tested by permutation of individuals between samples using FSTAT 2.9.3 [37].

### 2.5. Analyses of Signatures of Selection

#### 2.5.1. Standard Outlier Tests

Two different tests aimed at detection of loci subject to directional selection during domestication/captive breeding were used. Both approaches rely on the rationale that directional selection increases genetic differentiation between populations and reduces variability at linked loci. However, because both of the tests are based on different assumptions, identification of outlier loci simultaneously with both approaches provides additional support for the candidate status of a particular locus [29]. The first method, developed by Beaumont and Nichols [39] calculates *F*_ST_ for each locus in the sample, and coalescent simulations based on a symmetrical island model are used to generate data sets with the mean *F*_ST_ similar to the empirical distribution. To calculate approximate *P* values for each locus, 100,000 independent loci were generated and the simulated distribution of *F*_ST_ between two populations was then compared to the observed *F*_ST_ values, conditional on heterozygosity, to identify potential outliers as implemented in the software FDIST2. Sample sizes used to calculate *F*_ST_ from coalescent simulations were set to 16 individuals, corresponding to the empirical data. 

The second method to detect footprints of selection also identifies loci that exhibit extreme differentiation compared to the rest of the genome using the Bayesian likelihood method implemented via reversible jump Markov Chain Monte Carlo [40]. Using this approach, implemented in the Bayescan software, *F*_ST_ is modelled using a logistic regression model implementing a locus effect and a population effect, relaxing the assumption of symmetrical island model by allowing for population structure asymmetries. The estimation of the model parameters was automatically adjusted on the basis of short pilot runs (10 pilot runs with 5000 iterations each). We used a burn-in of 50,000 iterations and the total chain length of 500,000 iterations (thinning interval of 50 and sample size of 10,000) to identify loci under selection. Altogether, three independent runs were performed for each wild and domesticated/captive population pair. Bayescan estimates the posterior probability that a locus is under selection by calculating a Bayes factor, which is given by the ratio of posterior model probabilities of two models (selected/neutral), given the data. For example, a Bayes factor of two indicates that the data favor one model over the other at odds of two to one. According to Jeffreys' interpretation, Bayes factor between 3 and 10 (log10 = 0.5–1) is considered “substantial evidence” to favour one model over the other and corresponds to a posterior probability between 0.76–0.91. A Bayes factor between 10 and 32 (log10 = 1–1.5) is considered “strong evidence” of different statistical support for the two models and corresponds to a posterior probability between 0.91–0.97. However, this probability cannot be compared directly to the *P* value from FDIST2, since Bayescan explicitly takes all loci into account in the analyses. 

#### 2.5.2. Clustering of Loci with Extreme *F*_ST_ and lnRH Values

As a complementary approach to the outlier tests described above, we evaluated whether there is a tendency for adjacent loci to exhibit high differentiation (*F*_ST_) or large differences in genetic diversity (lnRH) [41] between wild and domesticated/captive population pairs. Such clustering of extreme loci would be indicative of genetic hitchhiking effects along a chromosome, while false positives from standard outlier tests are expected to occur randomly in the genome. Importantly, testing for clustering does not rely on specific assumptions about population history and thus provides complementary evidence to single-locus outlier tests. Specifically, we calculated the mean *F*_ST_ and mean absolute lnRH for pairs of markers along specific chromosomes and by using permutations evaluated whether the empirical pattern differs from random expectations (5,000 permutations). We subsequently identified locus pairs that exhibited elevated differentiation (*F*_ST_) or large differences in genetic diversity (lnRH), against the permutation-derived 95% significance threshold. Absolute lnRH was used as a parameter for quantifying the difference in genetic diversity (lnRH) between populations, as very short-term directional selection can either reduce or increase the genetic variation of a particular locus depending on the frequency of the allele linked to an advantageous mutation. For example, short-term directional selection initially increases the diversity when the positively selected (or linked) allele is at low frequency, while it reduces the variation when the positively selected (or linked) allele is at high frequency in the population. Loss of diversity measured as lnRH has been commonly used to identify outliers in microsatellite datasets but this statistic can also be applied to SNPs markers [42] as lnRH is not based on a particular mutation model [41].

#### 2.5.3. Estimation of False Discovery Rate (FDR)

To control for the multiple testing problem that arises when large numbers of markers are tested against the neutral null hypothesis, we employed the false discovery rate (FDR) methodology [43] implemented in the software *q* value to evaluate the proportion of putative outliers that are likely to be false positives (i.e., type I error); FDRs (*q* values) [44] were calculated for four neutrality tests based on the list of *P* values derived from (i) coalescent simulations (FDIST2 test), (ii) from expected normal distributions (lnRH test), and (iii) from permutation tests aimed at detecting clustering of loci with extreme *F*_ST_ and lnRH values. A bootstrap method was used for estimating the overall proportion of true null hypotheses as a more conservative approach compared to the *smoother *method as suggested by Storey and Tibshirani [43].

#### 2.5.4. Simulation of Selection from Standing Variation

To evaluate whether the observed patterns of genetic variation were compatible with the effect of strong directional selection, we used an individual-based forward-time simulation environment, simuPOP 1.0.2 [45], to explicitly simulate selection and hitchhiking processes based on the empirical data in hand. Specifically, we simulated selection on standing variation along the chromosome rather than from *de novo* mutations, as it is more likely that initial adaptation to an artificial environment occurs from standing variation. As a result, selection on preexisting genetic variation is expected to lead to immediate evolutionary response, fixation of more alleles of small effect and importantly, generally weaker footprints of selection compared to strong selective sweeps [46]. 

Given that the time since foundation of captive/domesticated strains are known and assuming equal population sizes and complete isolation (drift only) after the split, we calculated the effective population size using the estimated *F*_ST_ from *N*_*e*_ = *t*/(2*F*_ST_). We then constructed a simple single population split model applying the derived population genetic parameters for each population pair (IRL, gen. time 8, *N*_*e*_ = 330; SWE, gen. time 5, *N*_*e*_ = 65; CAN, gen. time 5, *N*_*e*_ = 238). We subsequently applied forward simulations using the empirical genotype data from wild population and genetic linkage map information to simulate the effect of selection along specific chromosomes on identified outlier loci. We first created an ancestral wild population by using empirical genotype data from the wild progenitor population (wild Burrishoole, Vindelälven, or Nashwaak) by multiplication of existing genotypes and subsequent random mating for three generations. After the split of the ancestral population, we simulated positive selection on the minor allele in a single candidate region per population pair. We assumed that the fitness of three genotypes AA, Aa, and aa in captive environment is 1, 0.7 and 0.4 (*s* = 0.6; *h* = 0.5), while there is no selection acting in the wild populations. Note that we are aware of only one-published single locus selection coefficient for Atlantic salmon (*s* = 0.49 on MHCII allele conferring resistance for *A. salmonicida*) [47] but selection coefficients of this magnitude and even higher have been observed in domesticated crops [48–50]. We subsequently let the populations evolve for five (SWE and CAN) or eight (IRL) generations according to the history of wild population-reared strain pairing and repeated the whole domestication/captive breeding simulation 25 times for each population pair. We then calculated the genetic differentiation between wild populations and reared strains using Weir and Cockerham's *F*_ST_ estimator [27], by sampling 16 individuals per population to evaluate whether artificial selection from standing variation can create comparable genetic differentiation patterns compared to the empirical data. We also validated our simulation procedure without implying selection (pure drift model) which produced genetic differentiation estimates similar to the empirical data (data not shown). The effect of selection on *F*_ST_ was visualized by creating box plots using SPSS 14.0.

## 3. Results

### 3.1. Summary Statistics

As expected, SNPs and microsatellites showed different levels of genetic variation (average gene diversity across populations 0.224 and 0.619, resp.; average number of alleles across populations 1.71 and 5.80, resp.; Mann-Whitney *U* test, all *P* values < 0.001). Contrary to earlier population genetic studies on natural and farmed Atlantic salmon populations [51–53], genetic diversity measures of wild and reared strain pairings did not differ significantly from each other (IRL and CAN; Wilcoxon signed ranks test, all *P* values > 0.05; Table 1) and for Swedish samples, both gene diversity and number of alleles were in fact higher in the reared strain compared with the wild population (Wilcoxon signed ranks test, both *P* values < 0.05; Table 1). Genetic differentiation, measured as *θ*, between reared strain/wild population pairs was rather low, ranging from ~0.01 in SWE and IRL to 0.039 in CAN but nevertheless, was highly significant (*P* < 0.001 for all three pairwise comparisons). When comparing wild population and reared strain pairs across different type of markers, the mean genetic differentiation estimates were rather similar (IRL: 0.017 versus 0.010; CAN: 0.031 versus 0.048; SWE: 0.012 versus 0.011 for microsatellites and SNPs, resp.), indicating that both types of markers are generally affected by a similar kind of evolutionary forces across the genome. Hardy-Weinberg disequilibrium was detected in 35 of 1370 loci by population tests at the 1% significance level before correction for multiple tests (data not shown). Approximately 14 of them are expected to emerge simply because of the large number of tests performed. 

### 3.2. Standard Outlier Tests

**Figure 1:**
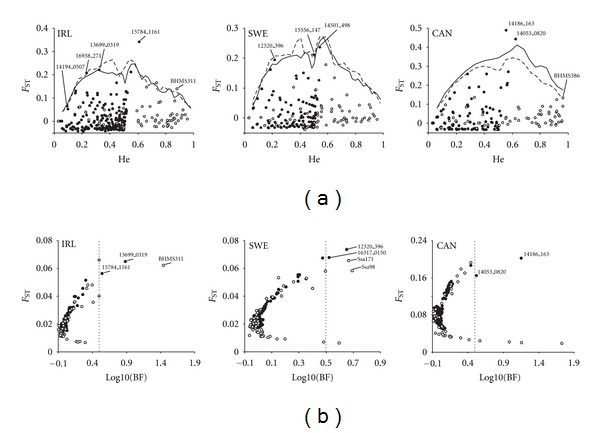
Results of two outlier tests in three independent pairs of wild-reared Atlantic salmon populations. Locus names of putative outliers potentially affected by selection (see Section 3) are indicated. (a) FDIST2: empirical distribution of *F*_ST_ against heterozygosity. The solid and dotted lines represent the upper 99% confidence interval for SNPs and tandem repeat markers, respectively; (b) Bayescan: The *F*_ST_ estimates plotted against Bayes factor. The dashed lines correspond to the Bayes factor 3 (log10(BF) = 0.5).

Altogether, eleven loci were identified as outliers in three wild-reared comparisons (three each in SWE and CAN and five in IRL) at the 99%  *P*-level using FDIST2 (Figure 1(a)). Those eleven loci consisted of nine SNPs and two microsatellite loci. Two outliers identified in SWE and CAN (loci 14053_0820 and 15556_147, resp.) were located on the same Atlantic salmon linkage group (LG23-Chrom. 16). The genetic distance between these markers in the female linkage map is 8 cM [28]. The Bayesian *F*_ST_ test (BAYESFST) identified nine outlier loci (three in IRL, four in SWE and two in CAN) with substantial evidence (Bayes factor > 3 corresponding to log10(BF) > 0.5) of directional selection according to Jeffreys' interpretation [27] (Figure 1(b)). Out of the fourteen outliers identified altogether, six loci were detected using both methods (Figure 1, Table 2).

### 3.3. Clustering of Loci

**Figure 2:**
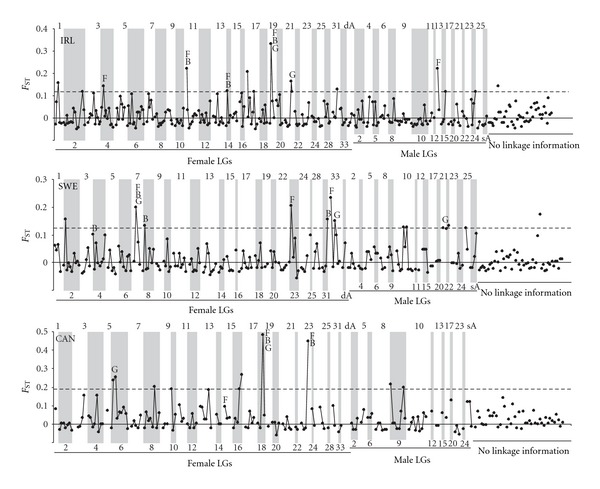
Distribution of *F*_ST_ along the female and male linkage groups (LGs) in three wild-reared Atlantic salmon pairwise comparisons (IRL, SWE, and CAN). F indicates markers that were identified as outliers at the 99% *P* level using FDIST2 [26]. B indicates markers that were identified as outliers (log10(Bayes factor) > 0.5) using Bayescan [27]. G indicates clustering of adjacent markers with elevated genetic differentiation (*F*_ST_) (permutation-based significance threshold, *P* < 0.05). Dashed lines correspond to the upper 2.5% of the empirical *F*_ST_ distribution and numbers along the *x*-axis indicate linkage groups as defined in [28].

**Figure 3:**
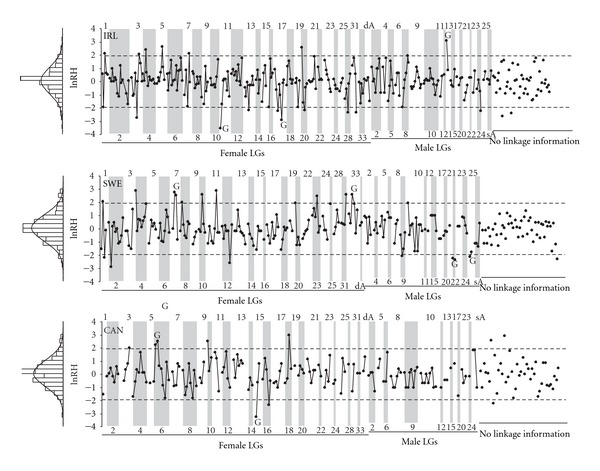
Distribution of lnRH along the female and male linkage groups (LGs) in three wild-reared Atlantic salmon pairwise comparisons (IRL, SWE, CAN). G indicates clustering of adjacent markers (permutation-based significance threshold, *P* < 0.05) that show large differences in genetic diversity measured as lnRH [29]. Dashed lines correspond to the 95% confidence limits (±1.96) of standardized lnRH estimates and numbers along the *x*-axis indicate linkage groups as defined in [28].

By utilizing the Atlantic salmon genetic linkage map information, we evaluated the evidence for clustering of loci that exhibit elevated genetic differentiation (*F*_ST_) or alternatively, show large differences in genetic diversity (lnRH) between wild-reared population pairs. Altogether, seven adjacent locus pairs (two in IRL, three in SWE, and two in CAN) exhibited evidence for clustering of markers (permutation-based significance threshold, *P* < 0.05) with high genetic differentiation (*F*_ST_) along the chromosome (Figure 2). However, in some cases high differentiation of adjacent markers was mostly driven by a single marker (IRL: LG19-Chrom. 8; SWE: LG7-Chrom. 24; CAN: LG18-Chrom. 23), while in other cases both loci exhibited elevated differentiation (IRL: LG21-Chrom. 26; SWE: LG22-Chrom. 17; CAN: LG6-Chrom. 12). When abs(lnRH) was used as a parameter to test the evidence for clustering of loci, nine adjacent locus pairs (three in IRL, four in SWE, and two in CAN) showed large changes in genetic diversity at 5% significance level (Figure 3).

### 3.4. False Discovery Rate (FDR) and Putative Candidate Regions

**Figure 4:**
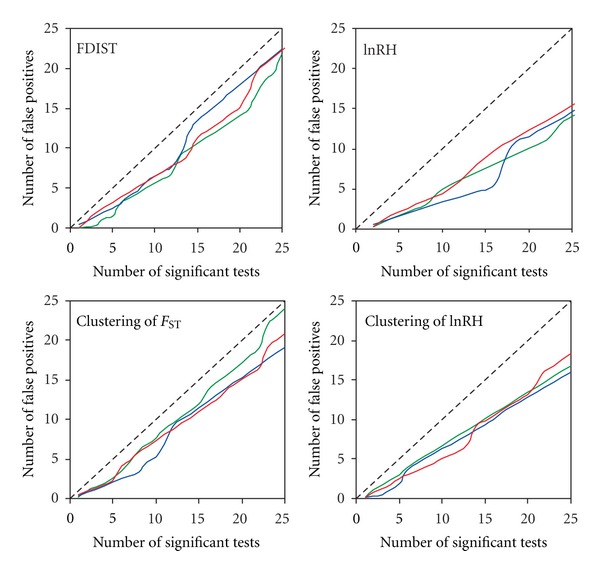
Estimated false discovery rate (FDR) for four different neutrality tests: FDIST2, lnRH, clustering of *F*_ST_ and lnRH. Green, blue and red lines correspond to *q* value estimates for three wild-reared Atlantic salmon pairwise comparisons (IRL, SWE and CAN, resp.).

To address the multiple testing problem that arises when large numbers of markers are tested against a null hypothesis, we estimated the false discovery rate (FDR) for the four previously-used neutrality tests: FDIST2, lnRH, clustering of adjacent loci with high *F*_ST_, or abs(lnRH). As expected, a considerable proportion of the loci that fall to the tails of the distribution are probably false positives, that is, they emerge because of the large number of tests performed (Figure 4). As a result, calculation of FDR revealed that if we select for subsequent validation of five loci based on extreme values from a single neutrality test, it is likely that from 2 to 4 loci out of five, depending on the particular population comparison and neutrality test, are likely to be false positives (type I error). On the other hand, the estimation of FDR also suggests that a small but nonneglible proportion of loci from our genome scan might be truly deviating from the neutral expectations and hence, represent potentially real footprints of directional selection related to domestication/captive breeding. When considering four different neutrality tests simultaneously, directional selection in a total of ten genomic regions was supported by multiple sources of evidence (Table 2). Among these ten putative candidate loci, four loci/genomic regions (IRL: LG11-Chrom. 3, 0–0.5 cM; IRL: LG19-Chrom. 8, 7.9–24.9 cM; SWE: LG7-Chrom. 24, 46.3 cM; CAN: LG18-Chrom. 23, 25.7–36.4 cM) were detected as outliers in three or more tests. None of the putative candidate regions overlapped in the three wild-reared population pairs.

### 3.5. Simulation of Selection and Genetic Hitchhiking

**Figure 5:**
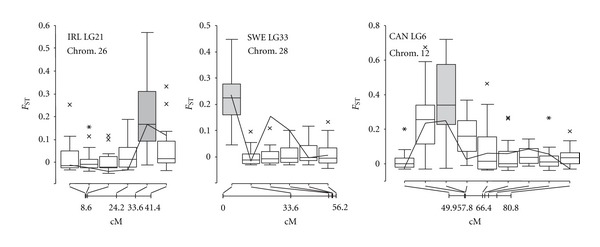
The effect of positive selection on standing variation on *F*_ST_ in three linkage groups (IRL: LG21-Chrom. 26; SWE: LG33-Chrom. 28, CAN: LG6-Chrom. 12). Bold line indicates the empirical distribution of *F*_ST_. Box plots showing the median and interquartile range, nonoutlier range, and outliers and extreme cases from 25 simulation replicates are shown with a line within the rectangle, rectangle, whiskers, cross and an asterisk, respectively. Filled grey rectangles correspond to the markers under selection (positive selection on minor allele); the position of each marker is provided on the *x*-axis.

We also evaluated whether strong directional selection over a very short period of time from standing variation also affects adjacent genetic markers along the chromosome, using the empirical genotype data from wild population as a source for subsequent forward simulations. We selected three linkage groups/chromosomes that contained previously-identified outlier loci (IRL: LG21-Chrom. 26, 15784_1161; SWE: LG33-Chrom. 28, 14501_498; CAN: LG6-Chrom. 12, 14186_163) with the closest adjacent marker 0 cM (CAN), 7.8 cM (IRL), and 33.6 cM (SWE) apart. Overall, strong selection on the minor allele resulted in high differentiation between wild population and reared strain pairs at selected markers, comparable with the empirical data (Figure 5). The hitchhiking effect of the adjacent markers, however, depended on the distance from the selected marker. For example, when the distance from the target of selection to the closest marker was relatively large (IRL: 7.8 cM; SWE: 33.6 cM), linked loci did not exhibit elevated differentiation between wild populations and reared strains. However, when markers were very closely linked to the target of selection, nearby loci also showed elevated differentiation (CAN: LG6-Chrom. 12). 

## 4. Discussion

In recent years, there has been a considerably increased interest in searching for signatures of natural or artificial selection, both in model and nonmodel organisms [54–56]. In the present study, we screened 331 codominant markers in three population pairs and identified ten genomic regions potentially affected by artificial selection associated with domestication/captive breeding in Atlantic salmon. We also evaluated how adaptation from standing variation affects adjacent SNP and microsatellite variation along the chromosomes and, by using forward simulations with strong selection, we were able to generate genetic differentiation patterns comparable to the empirical data. However, similar to other first-pass genome scans, the actual causative polymorphisms that affect the performance of individuals in the captive environment remain to be identified. As such, this study represents useful step towards identifying functional variants involved in domestication of Atlantic salmon. Below, we discuss our findings, their biological significance, and also methodological issues relevant for future hitchhiking-mapping studies. 

### 4.1. Overview of the Candidate Regions

By combining information from four different neutrality tests, in total, ten genomic regions showed signs of directional selection based on multiple sources of evidence (Table 2). When several linked markers exhibited evidence of directional selection, most of the identified candidate regions were relatively small ranging from zero to a few centimorgans (cM) in the female Atlantic salmon linkage map. In two cases, the adjacent outliers were located in the same contig [28] less than 1 kb from each other (IRL: LG13-Chrom. 19; SWE: LG22-Chrom. 17). In three cases, the putative candidate regions were larger, ranging from 10 to over 50 cM (CAN: LG18-Chrom. 23; IRL: LG19-Chrom. 8; SWE: LG33-Chrom. 28). These regions may represent false positives or alternatively contain multiple independent targets for selection, as our simulations indicate that the signatures of selection from standing variation do not necessarily extend over such large chromosomal regions (Figure 5). Examination of the genes within candidate regions revealed that two outlier loci in SWE LG33-Chrom. 28, corresponding to cystatin precursor and brain protein 44-like protein, were also reported in [24] as being among 1.7% of genes showing differential expression between the progeny of farmed and wild Atlantic salmon. Assuming that 3557 genes analysed by Roberge et al. [24] had an equal chance of ending up in our candidate list presented in Table 2, such overlap would be rarely emerge by chance alone (10,000 permutations, *P* < 0.012). Interestingly, the same two genes also showed significant gene expression differences between domesticated- and wild-rainbow trout (*Oncorhynchus mykiss*) [57]. These findings, combined with the gene expression information, indicate that cystatin precursor and brain protein 44-like-protein might be interesting targets for more detailed genomic analysis.

We found that several of our candidate regions overlap with Atlantic salmon QTLs identified for various ecologically and economically important traits in earlier studies. For example, linkage groups 6 (Chrom. 12), 7 (Chrom. 24), and 11 (Chrom. 3) have been shown to harbour QTLs for body weight [58, 59], condition factor [58, 60], precoccious male parr maturation [Pederesen et al. unpublished], morphometry [61], and time of emergence [62]. Similarly, other linkage groups that included regions influenced by domestication have been shown to harbour QTLs for body weight (LG14-Chrom. 21; LG18-Chrom 23, LG19-Chrom.8, LG23-Chrom. 16), length (LG13-Chrom.19), LG14-Chrom. 21, LG18, LG19), condition factor (LG13-Chrom.19, LG18, LG23-Chrom. 16), and fillet color (LG 11-Chrom. 3, LG13-Chrom. 19, LG19-Chrom. 8) [58–62]. Therefore, it is possible that some of the identified regions under selection are associated with growth, morphology, and life history variation. However, it is important to remember that Atlantic salmon males show an extremely low recombination rate across the vast majority of the genome [31] and many of the QTLs have been identified using a male as a mapping parent. Hence, considerable uncertainty exists about the exact location of many quantitative trait loci in Atlantic salmon (but see also [63]). It is noteworthy that one of our outlier SNPs (LG11-Chrom. 3, 314711_157) has been identified as a strong outlier under diversifying selection among the North American Atlantic salmon populations [64]. 

### 4.2. Domestication in Salmonid Fishes: Same Pathways, Different Genes?

Our study provides little support for the hypothesis of independent parallel genetic changes at the DNA level in Atlantic salmon from different population pairs as none of the identified candidate regions overlapped in the three wild-population/reared strain pairs. This is in contrast with earlier studies at the gene expression level that have demonstrated the presence of parallel differences in transcription of genes between independent domesticated- and wild-Atlantic salmon [24]. However, a closer examination of the transcription patterns reveals that only a relatively small proportion of differentially expressed genes (16%) exhibited parallel changes in independent farmed reared strains, while the majority of changes in gene transcription are strain specific. Similarly, in rainbow trout (*Oncorhynchus mykiss*) and brook charr (*Salvelinus fontinalis*), differentially expressed genes between reared strains and wild populations have been shown to belong primarily to similar biological functions such as energy metabolism of lipids and protein synthesis, but the specific genes involved in these biological functions were found to be different [57, 65]. These results are also in accordance with the recent genome scan by Flori et al. [1] who found that the same physiological pathways but different genes are affected by artificial selection in dairy cattle. Such parallel effects at higher functional levels in domesticated animals strongly suggest that the same downstream pathways are being affected during the domestication process. As a result, studies focusing on analyses of gene expression alone provide only limited information about the number (and position) of causative allelic variants responsible for the rapid changes in gene expression of domesticated fish. This has been elegantly demonstrated by Devlin et al. [66] who showed how the insertion of growth hormone gene can generate whole genome transcriptome signatures very similar to the domesticated strain in coho salmon (*Oncorhynchus kisutch*). 

It is also possible that several other factors contribute to the population-specific signatures of selection, such as (i) different selective regimes in farmed versus ranched strains, (ii) different selective regimes between separate ranched strains, or, (iii) allele frequency differences between populations/strains. In fact, it is possible that all of these factors contribute to the lack of overlap of outliers because (i) farmed strains experience strong intentional selection for growth, disease resistance, delayed maturity, and other commercially important traits while ranched strains experience inadvertent selection in captivity as well as natural selection at sea [22, 67]; (ii) the selection regimes at the feeding grounds of the Atlantic Ocean (IRL) and the Baltic Sea (SWE) may be different; and (iii) loci that are polymorphic among European salmon are often not variable in the North American populations and *vice versa*; even if polymorphism exists in all strains, when the allele that is linked to the positively selected site is already at high frequency in a particular population, selection does not necessarily induce a strong increase in the level of genetic differentiation and reduction in diversity. 

### 4.3. Limitations and Future Directions

There is growing consensus that such genome scans typically suffer from low power and high-false positive rates [68–71]. As a consequence, it is important to note that our analyses, and hence interpretations, are subject to several limitations. First, our study is based on a relatively small number of individuals, which affects the accuracy of allele frequency estimates. Therefore, confirming the allele frequency estimates using larger sample sizes and multiple cohorts (including multiple tributary populations from the Saint John River) could be clearly beneficial. However, the forward simulations were able to generate strong footprints of selection when using similar numbers of individuals, thus suggesting that our study indeed have reasonable power. The variable outcome of individual simulations, however, might be at least partially influenced by the limited sample size. The limited sample size may have also some effect on the genetic diversity estimates. For example, in contrast to many earlier studies that have documented lower variability in farmed Atlantic salmon compared to wild populations (e.g., [51–53]), we did not observe lower levels of genetic diversity in reared strains compared with their wild progenitor populations. However, earlier studies have compared the diversity of wild populations with farmed Norwegian salmon which have the longest history of selective breeding [52, 53]. Therefore, it is not unexpected that reared strains of Burrishoole (IRL) and Umeälven (SWE) exhibit relatively similar levels of genetic variation compared to their wild counterparts, especially considering the relatively large number of fish used during breeding in recent years (pers. comm. P. McGinnity; J. Nilsson). This is also in accordance with the study of Vasemägi et al. [72] who reported relatively similar levels of genetic diversity in captive Umeälven and wild Vindeläven salmon. 

Second, even though the number of codominant markers used in this study is higher than typically applied in nonmodel organism genome scans to date, it still covers only a very small proportion of the Atlantic salmon genome. For example, Pollinger et al. [73] estimated that for detecting strong recent artificial selection on novel mutation in dog, one would require a spacing of one highly polymorphic marker per 0.8 centimorgans (cM) or less. Assuming that the length of the female Atlantic salmon linkage map is close to 2000 cM [28], then approximately 2500 evenly spaced markers would be needed to cover the Atlantic salmon genome at this density. Therefore, a recently developed high density SNP chip in Atlantic salmon consisting of more than 5500 SNPs should provide a valuable tool for mapping regions under selection in the future [31]. On the other hand, it has been suggested that as many as 150,000 SNPs will be required for identifying genome-wide footprints of selection in the bovine genome which has the genome size comparable to Atlantic salmon [74]. To this end the use of next generation sequencing and genome complexity reduction methods hold great promise to gain whole-genome coverage for population genomic studies even for nonmodel species [11, 75]. The other alternative strategies for hitchhiking mapping include focusing on *a priori* defined different functional groups or pathways rather than single genes [76–78]. Finally, several major chromosomal differences between the European and North American Atlantic salmon have been recently demonstrated while the fine-scale order of genetic markers has been conserved [79]. Hence, our analysis using genetic maping information should be taken with caution until the sequence of the Atlantic salmon genome becomes available.

In conclusion, our results together with other recent studies [1, 11, 80, 81] illustrate the significance of standing genetic variation during the early stages of adaptation in leading to rapid evolutionary responses to novel environments. Consequently, one of the future challenges for hitchhiking mapping studies is to develop methods and approaches that consider alternatives to the classic theory of “hard” selective sweeps (adaptation from *de novo* mutation) in order to obtain more comprehensive understanding about molecular mechanisms of adaptation. Species with domesticated/captive strains are likely to remain popular models in this context, providing unique opportunities to identify molecular targets of artificial selection. 

## Supplementary Material

Marker and genetic map information of studied SNP and microsatellite loci.

## Figures and Tables

**Table 1:** Diversity indexes of the Atlantic salmon populations included in the study.

Pop.	Origin	Mean gene diversity			Mean number of alleles		
		Microsatellites (range)	SNPs (range)	All	Microsatellites (range)	SNPs (range)	All
IRE	Wild	0.661 (0–0.958)	0.319 (0–0.525)	0.385	6.08 (1–18)	1.90 (1-2)	2.71
	Reared	0.648 (0.063–0.944)	0.321 (0–0.523)	0.384	5.52 (2–17)	1.92 (1-2)	2.62

SWE	Wild	0.562 (0–0.948)	0.224 (0–0.542)	0.294	4.91 (1–15)	1.68 (1-2)	2.36
	Reared	0.587 (0–0.984)	0.233 (0–0.524)	0.307	5.23 (1–20)	1.72 (1-2)	2.45

CAN	Wild	0.638 (0.063–0.951)	0.125 (0–0.524)	0.219	6.73 (2–15)	1.54 (1-2)	2.50
	Reared	0.621 (0–0.963)	0.125 (0–0.533)	0.216	6.32 (1–20)	1.50 (1-2)	2.39

Pop.: population.

**Table 2:** Ten loci/genomic regions supported by multiple lines of evidence of being affected by directional selection during domestication/captive breeding in Atlantic salmon. Microsatellite markers are given in italics.

Pop.	Linkage group (d—dam, s—sire)	Chromosome	Markers	Map position cM	FDIST2 *P* < 0.01	BayeScan log10(BF) > 0.5	Clustering of loci		Annotation^a^
							*F*_ST_ *P* < 0.05	lnRH *P* < 0.05	Top hit
	d11	3	13699_0319	0	X	X		X	
			14711_157	0.5				X	Q5R5Y3∣B3GT2_PONPY Beta-13-galactosyltransferase 2 (Beta-13-GalTase 2) (Beta-3-Gal-T2)
	d19	8	15784_1161	7.9	X	X	X		Q61425∣HCDH_MOUSE Short chain 3-Hydroxyacyl-CoA dehydrogenase mitochondrial
IRL			*BHMS365 *	24.9			X		
	d14	21	*BHMS311 *	1.5	X	X			
	s13	19	16938_271	25.9	X			X	gnl∣CDD∣29599 cd01417 Ribosomal_L19e_E Ribosomal protein L19e eukaryotic. L19e
			16938_888	25.9				X	gnl∣CDD∣29599 cd01417 Ribosomal_L19e_E Ribosomal protein L19e eukaryotic. L19e

	d07	24	12320_396	46.3	X	X	X	X	sp∣P20135∣GSTT1_CHICK Glutathione S-Transferase theta-1 (GST-CL1) (GST class-theta)
			*SSsp2216 *	46.3			X	X	
	s22	17	12302_129	7			X	X	sp∣Q9JJL3∣SO1B2_MOUSE Solute carrier organic anion transporter family member 1B2
SWE			12302_196	7			X	X	sp∣Q9JJL3∣SO1B2_MOUSE Solute carrier organic anion transporter family member 1B2
	d33	28	14501_498	0	X				
			16532_248	52.5			X	X	sp∣P63030∣BR44L_MOUSE Brain protein 44-like protein
			12035_0440	54			X	X	sp∣Q98967∣CYT_ONCKE Cystatin precursor

	d18	23	14186_163	25.7	X	X	X		sp∣Q96KP1∣EXOC2_HUMAN Exocyst complex component 2 (Exocyst complex component Sec5)
			*Ssa85DU *	36.4			X		
CAN	d06	12	16937_940	57.4			X	X	sp∣P24844∣MLRN_HUMAN Myosin regulatory light chain 2 smooth muscle isoform (Myosin RLC)
			17580_1116	57.8			X	X	sp∣Q6NWF6∣K2C8_BRARE Keratin type II cytoskeletal 8 (Cytokeratin-8) (CK-8) (Keratin-8)
	d23	16	14053_0820	33.4	X	X			

^a^BLASTX search against CDD and Swissprot databases [28].
